# Keloids as a rare complication of voluntary medical male circumcision: findings from PEPFAR’s notifiable adverse events reporting system

**DOI:** 10.1186/s12894-025-01949-7

**Published:** 2026-01-16

**Authors:** Stephanie Davis, Eniko Akom, Valerian Kiggundu, Sarah Nabukera, Anne Thomas, Daimon Simbeye, Oscar Ernest Rwabiyago, Suzan Mmbando, Alick Kayange, Neway Fida, John Mandisarisa, Tungamirai Mhuka, Wezi Msungama, Martin Kapito, Faustin Matchere, Martin Maulidi, Elijah Odoyo-June, Leonard Soo, Ambrose Juma, Todd Lucas

**Affiliations:** 1 Division of Global HIV and Tuberculosis, United States Centers for Disease Control and Prevention, Atlanta, GA USA; 2 Henry M. Jackson Foundation for the Advancement of Military Medicine, Inc., Bethesda, MD USA; 3 U.S. Military HIV Research Program, CIDR, Walter Reed Army Institute of Research, Silver Spring, MD USA; 4 Global Health Consultant, Washington, DC USA; 5 Office of Program Quality, The U.S. President’s Emergency Plan for AIDS Relief (PEPFAR), Bureau of Global Health Security and Diplomacy (GHSD), Washington, DC USA; 6 U.S. Department of Defense HIV/AIDS Prevention Program, Defense Health Agency, San Diego, CA USA; 7 Division of Global HIV and Tuberculosis, United States Centers for Disease Control and Prevention, Dar es Salaam, Tanzania; 8 Ministry of Health, Dar es Salaam, Tanzania; 9 United States Department of Defense, Dar es Salaam, Tanzania; 10 United States Agency for International Development, Dar es Salaam, Tanzania; 11 Division of Global HIV and Tuberculosis, United States Centers for Disease Control and Prevention, Harare, Zimbabwe; 12 Zimbabwe Technical Assistance, Training and Education Center for Health (ZIMTTECH), Harare, Zimbabwe; 13 Division of Global HIV and Tuberculosis, United States Centers for Disease Control and Prevention, Lilongwe, Malawi; 14 Ministry of Health, Lilongwe, Malawi; 15 United States Department of Defense, Lilongwe, Malawi; 16 United States Agency for International Development, Lilongwe, Malawi; 17 Division of Global HIV and Tuberculosis, United States Centers for Disease Control and Prevention, Nairobi, Kenya; 18 United States Agency for International Development, Nairobi, Kenya; 19 Ministry of Health, Nairobi, Kenya; 20 HIV Prevention Branch, Division of Global HIV and Tuberculosis, Department of Health and Human Services (HHS), U.S. Centers for Disease Control and Prevention (CDC), Atlanta, USA

**Keywords:** Circumcision, Keloids, HIV prevention, Adverse events

## Abstract

**Background:**

Keloids are rare but challenging complications in penile surgeries, including circumcision. The President’s Emergency Plan for AIDS Relief (PEPFAR) supports voluntary medical male circumcisions (VMMC) for HIV prevention in sub-Saharan Africa, and has monitored major adverse events through its Notifiable Adverse Event Reporting system (NAER) since 2015, providing unique opportunities to understand keloid epidemiology and risk factors.

**Methods:**

All 2015–2023 NAER keloid cases were reviewed. Variables abstracted included age at VMMC, time course, and case characteristics. Descriptive analysis and Fisher’s exact test of the association between client age (< 15 years vs. ≥ 15 years) and keloids were performed. Systematic literature searches were also conducted on case reports, outcomes and management guidelines, to produce a narrative summary and comparisons between the NAER cases and prior literature.

**Results:**

Eleven cases were reported. Clients were < 15 years old in 10/11, with median age 12 years and mean time to diagnosis 1.8 years. Clients aged 10–14 years had a significantly higher risk for keloids compared to those aged 15 and older (*p* = 0.0004). Case characteristics were similar to those in prior case series, as was management, except that silicone gels or sheeting were not used. Follow-up was too brief to assess outcomes.

**Conclusions:**

Keloids were a rare complication of PEPFAR-supported VMMCs. Keloids were strongly associated with age 10–14 years at circumcision as compared to older age. Programs can remind clients to return to VMMC sites even for late complications, and develop expert referral networks. Silicone gels or sheeting could represent an additional conservative management option.

**Supplementary Information:**

The online version contains supplementary material available at 10.1186/s12894-025-01949-7.

## Introduction

Male circumcision decreases men’s risk of acquiring HIV through sex with women by about 60% [1–3]. For this reason, voluntary medical male circumcision (VMMC) is a World Health Organization-recommended public health intervention in countries with generalized HIV epidemics and low male circumcision prevalence. Through 2024, these priority countries, all in sub-Saharan Africa, have provided over 37 million VMMCs [4, 5], mostly supported by the President’s Emergency Plan for AIDS Relief (PEPFAR).

While major adverse events (AEs) are rare, safety remains crucial. PEPFAR and national governments have developed reporting practices to detect and investigate such AEs and make system changes to decrease future risks. PEPFAR’s Notifiable Adverse Events (NAE) Reporting (NAER) seeks to capture all AEs meeting its NAE criteria among all countries conducting PEPFAR-supported VMMCs. This database has independent value as a large prospective international dataset capturing hundreds of circumcision AEs [6], and has identified patterns that prompted further investigations of several AE types [7–9] leading to PEPFAR policy changes to enhance safety. However, it has not been systematically studied for such patterns with respect to keloids, a rare but serious complication.

Keloids, abnormal scar tissue proliferations extending past the original wound borders, consist of dense benign collagen matrices containing multiple cell types [10]. On the penile corona, they can interfere with sexual activity and cause itching or pain with daily activities due to friction with clothing. Analyzing the unique NAER dataset provides an opportunity to identify keloid risk factors and any program gaps, in order to minimize keloid risk for VMMC clients. In addition, by substantially increasing the total cases in the sparse literature on this rare complication, such an analysis could benefit penile surgical practice more broadly. Here, we report on NAER keloid cases contextualized by a brief systematic review which draws heavily from other recent reviews [11, 12] but with an expanded global scope.

Finally, before and during NAER’s operational years (2015-present), several policy changes took place which affect data completeness and interpretation:

- In 2014, PEPFAR policy was changed to require use of a specific surgical method termed “dorsal slit” instead of the previously common “forceps-guided” method in clients aged under 15 years or with immature genitalia, to prevent glans injuries [13].
- Before 2015, reporting of serious adverse events to PEPFAR was encouraged on an ad-hoc basis for PEPFAR-supported programs. In 2015, it became mandatory.
- In 2020, PEPFAR discontinued funding surgical VMMC for boys aged under 15 years, with rare exceptions,^1^ based on emerging evidence of increased risk for glans injuries and urethral fistulas compared to clients aged 15 years and older. Previously, these boys were a major source of VMMC uptake, accounting for 41-48% of all PEPFAR-supported VMMCs during 2015–2019 [14, 15].

## Materials and methods

### NAER data

 The qualifying criteria for mandatory NAE reporting are: 1: any of: death; complete or partial amputation; tetanus, any adverse event resulting in at least probable permanent disability or anatomic deformity; displacement in the case of circumcision devices that remain in-situ19; or hospital admission for 3 or more days; and 2: occurring within 30 days of VMMC regardless of perceived association with the circumcision, or any time after 30 days if possibly associated (keloids would typically meet the latter part of this criterion).

Reporting is done via a series of standardized forms documenting an active investigation process led by PEPFAR field staff that can include site visits, provider interviews, chart review and patient follow up. Fields include key characteristics of the client, the site, the procedure, and the AE including workup and management (see below). Our database access was continual through the time of submission, but documentation of an individual NAEs submitted to PEPFAR typically ends when definitive management is considered to be finished.

Cases reported from January 2015 to September 2021 were identified by having a keloid documented in the NAER tracking spreadsheet used till that time, and cases reported from October 2021 (after conversion to electronic reporting) until September 2023 were identified by reviewing all electronic reports for mentions of keloid in any electronic text field. Reports retrieved were reviewed to eliminate any in which keloids were not the ultimate diagnosis. From included reports, we abstracted the variables appearing in Table 1. Variable value categories and conventions included:

- For known risk factors: family history of keloids, keloids on physical exam
- For circumcision technique: forceps-guided, dorsal slit or device
- For procedure site type: static (a fixed facility where VMMC consistently available), outreach (a site where VMMC intermittently is available), mobile (a portable facility such as an operating theater trailer)
- For client age at time of VMMC: when not provided explicitly at time of VMMC, we used age in NAE report
- For time from VMMC to first symptoms and from VMMC to diagnosis: the midpoint was used if only a range was provided
- For symptoms: “itching”, “pain” (including discomfort) and others

Descriptive analysis was performed. Fisher’s exact testing was performed on the association between client age (< 15 years vs. ≥ 15 years) and keloid NAEs reported per VMMC performed, based on the subset of reported keloids that resulted from procedures performed during Jan 2015-September 2023^2^ and numbers of VMMCs performed in the same period with client ages reported, according to data from routine required PEPFAR reporting (Monitoring, Evaluation and Reporting indicators, or MER data) [13]. Other potential predictors were not tested statistically due to missingness (e.g., suture type) or imbalanced data (e.g. no clients had intraoperative complications). No interpolation was done.

### Literature review

We used two independent librarian-designed systematic literature searches, one for reports on epidemiology of post-surgical penile keloids and one for penile keloid management, without restrictions on time period, geography, or study type, through February 24, 2024. Databases included Medline, OVID Global Health, CINAHL, Scopus, Africa-Wide Information, and the World Health Organization Global Index Medicus. Please see Appendix 1 for the full search strategy. Inclusion criteria for all articles included being available in English or translatable via Google Translate. Article on keloid epidemiology were also required to describe keloid or other scarring problems resulting from any penile surgery including, but not limited to, circumcision. Those on keloid management were required to describe management of keloids or other scarring problems resulting from any penile surgery (not injury); review and guidance articles were not excluded.

Articles passing these initial screenings were subjectively full-text reviewed for unique contributions to a narrative review to contextualize the NAER data, and objectively reviewed for unique case reports to be included in our assessment of the total number available in previous literature. All screening was done by one investigator (SD).

For epidemiology, we described included articles in a narrative summary organized by subtopic and clinical context. For treatment, we compared included articles against those summarized in two reviews of penile keloids identified through the literature search, Alzeerelhouseini et al. [10] and Isolde et al. [11] (These reviews used, respectively, unspecified and potentially less-global search strategies). Finally, we searched results for relevant medical specialist society keloid management guidelines, also adding UpToDate and Pubmed guideline searched for 2014–2024, which we also captured in a narrative summary.

This work was determined by CDC to constitute a non-research usage of routinely-collected program (NAER) data.

## Results

### NAER data

Table 1 provides a line-list of the 11 keloid NAEs reported in VMMC clients since 2015 (regardless of the year of circumcision) with key variables. There was considerable missingness among variables. Notable summary observations included:

- All but one keloid developed in clients aged < 15 years at VMMC.
- All reports came from four of 15 VMMC implementing countries.
- Mean time from VMMC to diagnosis was 1.8 years.
- Among 6 cases providing timing of first symptoms, delay from VMMC till first symptoms was typically one year (not shown).
- Keloids typically developed at the wound site; one exception developed at the penile base, presumably an anesthetic injection site. Keloids encompassed ≥ 50% penile circumference in 6 of 8 cases reporting anatomic extent.
- Excision was the typical management (9 of 10 cases with management determined), always with intra- and/or post-operative steroids, but not often (2/9 excision cases) with preoperative steroids.
- At last followup, two excision cases had unknown outcomes, three had recurrence with further management planned, and four were healing well at ≤ 4 months or unknown followup duration.
- Nine keloids were included in statistical testing, among 7,368,596 VMMCs in clients aged < 15 years and 17,161,514 in clients aged ≥ 15 years. Age had a highly significant association with reported keloid cases per million VMMCs performed, at *p* = 0.0004, with 1.09 vs 0.05 keloids reported for every million VMMCs performed among clients aged < 15 years and ≥ 15 years respectively.

**Table 1:** Characteristics and clinical courses of PEPFAR voluntary medical male circumcision clients in southern and eastern Africa with postsurgical penile keloids reported to Notifiable Adverse Event Reporting system in 2015–2023

**Year of VMMC**	**Country**	**Family or personal history of keloids or keloids noted on exam**	**Intra- or post-operative complications**	**Client age at VMMC in years**	**Years from VMMC to keloid diagnosis**	**Symptoms reported**	**Location (if not wound site) and % circumference involved**	**Management and outcome**
2011	Tanzania	none	none	14	2.9	pruritis, pain	at penile base ≥ 50%; at circum-cision site < 25%	**1st recurrence** (2 years later) – pre-operative steroid injections and excision; outcome—recurred **2nd recurrence** (3 years later) – steroid injections without effect, referred to specialist with management pending; outcome—not documented
2013	Zimbabwe	none	none	12	2.7	pain	≥ 50%	**Initial**—excision with post-operative steroid injections; outcome—healing well at 1 month postop
2016	Malawi	not provided	none	12	1.9	pruritis	≥ 50%	**Initial**—excision with intra-operative steroid injections; post-operative steroid injections planned; outcome—healing well at 1 week post op
2016	Zimbabwe	none	none	13	0.9	not provided	not provided	**Initial**—referred to specialist; management decision pending; outcome—not documented
2016	Zimbabwe	none	none	13	1.5	not provided	< 50%	**Initial**—excision with intra-operative and post-operative steroid injections; outcome—not documented
2016	Zimbabwe	none	none	10	1.9	pruritis	< 50%	**Initial**– excision with pre- and intra-operative steroid injections; outcome—recurred **1st recurrence**(1 year later) – excision with pre- and intra-operative steroid injections; outcome—recurred **2nd recurrence**(1 year later) – Referred to specialist, management unknown; outcome—unknown
2016	Kenya	No FH keloids, personal history not provided	Not provided	12	1.6	asymptomatic	≥ 50%	**Initial**—excision with intra- and post-operative steroid injections; outcome—well-healed at 4 months postop
2017	Zimbabwe	none	none	11	1.4	not provided	Not provided	**Initial**—steroid injections; outcome—unknown
2018	Zimbabwe	none	none	20	1.3	not provided	not provided	**Initial**—excision with intra- and post-operative steroid injections; outcome—unknown
2018	Kenya	no FH keloids, small hypertrophic scar on exam	Not provided	11	1.2	asymptomatic	≥ 50%	**Initial**—excision with intra-operative steroid injection; outcome – diagnosed with incomplete excision and referred to plastic surgeon who completed excision at later date; outcome—reported as “complete recovery” with unknown follow up time
2019	Kenya	none	none	10	2.5	pruritis	interrupted (multiple nodules) but > 50% circumferential in total	**Initial**—single steroid injection without follow-up; one year later, excision with intra- and post-operative steroid injections; outcome—recurred Recurrence (1 year later)—with management pending; outcome—unknown

### Literature review

#### Epidemiology of keloids and other scar problems after male circumcision

In total, 271 articles were identified across all search strategies, of which 151 were excluded in the title screening, virtually all due to lacking primary data. Of the remaining 120 articles, on full-text review, 13 (cited in this section) contributed subjectively unique data on keloid epidemiology, risk factors and/or management, in addition to the systematic reviews [11, 12].

Keloid incidence data, with any denominator of at-risk males, were not found. When including non-keloidal hypertrophic scarring (limited to the wound borders) after circumcision, nearly all data found applied to circumcision methods no longer in use [16], not in standard use [17], or representing a small minority of circumcisions (ShangRing, a device-based sutureless method) [18], giving them questionable generalizability. Two exceptions reported 2–3% prevalence of hypertrophic scarring [19, 20]; one other, severely limited by its online recruitment strategy [21], obtained a 34% self-reported prevalence of hypertrophic scarring among circumcised adult Taiwanese.

Although multiple characteristics (family or personal keloid history, darker skin, infection, prolonged healing, rough tissue handling, excessive wound tension) [10, 11, 22] are commonly proposed as risk factors, our search did not identify primary data supporting these or any other characteristics as risk factors.

Table 2 compares descriptive findings from NAER data with those in the literature reviews, where at least one review reported the same variable. Client age and time from procedure to keloid development were similar across sources. NAER cases rarely had a positive personal keloid history, while nearly a third of cases reported by Alzeerelhousseni et al. [11] did. NAER post-treatment followup was several months at most, where the other reviews often reported over a year.

**Table 2:** Comparison between PEPFAR NAE keloid cases reported 2015–2024 and keloid cases without date restriction identified in two literature reviews on key characteristics, among those with characteristic-specific data

Characteristic	PEPFAR NAE data: *n* = 11	Isolde et al (penile keloids through 2023): *n* = 19	Alzeerelhouseini et al. (penile keloids through 2022): *n* = 34
% resulting from circumcision	100%	57%	73.5%
Client age (years)	Median 11	Mean 11, median 8	Mean 12
Breakdown of client ethnicity	Of 11: 11 (100%) sub-Saharan African (southern and eastern)	(not reported)	Of 29: 12 white, 9 sub-Saharan African, 5 Asian (southern or eastern), 3 other
Any personal keloid history	Of 10: 1 (10.0%)	(not reported)	Of 29: 9 (31%)
Time elapsed from procedure to keloid developing	Median 1 year; range.3—2 years	Mean 0.7 years, median 0.5 years; range 0.4–2 years	(not reported)
Length of follow up after treatment	1 week-3.5 months	2.5 ± 1 year	Median 1 year (5 months-3 years)^*^

^*^Length of follow up data provided only for cases without recurrence

#### Treatment

##### Published case studies and NAER cases

With respect to post-circumcision keloids, our search identified three additional publications [9, 23, 24] encompassing four additional cases, for a total of 38 cases in the literature, and one retrospective on keloids following a variety of penile procedures [25]. No new publications contributed unique treatment approaches.

Otherwise, the two systematic reviews reported similar distributions of treatment approaches. Surgical excision was most common, alone, with intraoperative steroids, or with postoperative steroids. Intralesional steroid injection was a somewhat less common first approach. Radiotherapy, compression and silicone gel or sheet occlusion were almost always used as post-excision adjunctive therapy. Treatment of NAER cases (Table 1) was similar except for these adjuncts.

##### Society guidelines

Guidelines on keloid treatment included multiple options: silicone-based products, pressure dressings, hydrocolloid dressings, topical and/or injectable agents (e.g., steroids, antineoplastic drugs, calcium channel blockers, retinoid acid, botulinum toxin), cryotherapy, radiation therapy, variations of laser therapy, and surgery (including complete and subtotal resections) with variations on closure techniques. Recommendations specifically for penile keloids were lacking.

Recurrent themes were 1) taking an individualized approach to patients based on clinical history and anatomic location, size, appearance, and age of the keloid, 2) starting with more conservative interventions, and 3) reaching agreement with the patient on therapeutic goals and how successful progress towards those goals should be measured.

## Discussion

This analysis adds 11 case reports of keloid development following circumcision to the 38 we found already published. Because of the unique inclusion of denominators, we believe it is the first to assess the frequency of this complication (9 among the approximately 25 million circumcisions supported by PEPFAR between January 2015 and September 2023) in an African population, often considered to be at elevated risk [10, 11], and the first to test the hypothesis that youth have an elevated risk for keloid formation after circumcision as compared to older clients.

This apparent age-related risk could be a true finding, resulting from either biological predisposition [26] and/or age-related behavioral factors like diligence in wound hygiene or avoiding reinjury during healing. It could also be an artifact of higher care-seeking rates among youth, and/or of more successful exclusion of keloid-predisposed older clients than younger clients from VMMC through screening (as they would be more likely to have a prior keloid history to report, and to know their family history). The fact that no keloids were reported after PEPFAR largely discontinued circumcising clients less than 15 years old in 2020, despite no change in NAE reporting criteria, may provide further support for a true age-related keloid risk, though the absolute risk of reported keloid remains very low across age groups.

We did not identify other modifiable risk factors in any case but one (missed personal history). With respect to the other potential risk factors—suture type, clinical setting, surgical method—data imbalance and/or missingness prevented reaching conclusions. Most NAER keloids were ≥ 50% circumferential, perhaps suggesting the importance of factors which could affect the full wound surface, such as genetic predisposition and tissue handling or tension.

Nearly all NAER cases were managed with injected steroids and local excision. Published case studies might be considered biased by a tendency to capture cases requiring surgical management, but NAER approaches were similarly surgery-led (compared to the guidelines, which recommended conservative-first approaches). In fact, no NAER cases were managed using the conservative alternatives in the guidelines, e.g., compression therapy, silicone gel or sheeting, and in one case radiotherapy. In PEPFAR settings, radiotherapy would vary in availability, but silicone gels or sheets could be a noninvasive, benign initial option. Anecdotally, authors based in southern and eastern Africa find these are not in routine use. Availability may pose a challenge but could be explored.

The lack of rigorous evidence precludes more definitive therapeutic recommendations. Adhering to the principles in existing guidance is prudent, especially referral to an experienced provider or multi-disciplinary team rather than attempting excision without experience, and consideration for a trial of conservative management.

In the narrative literature review, the lack of keloid incidence and statistically testable risk factor data most likely reflects their delayed formation, long after post-circumcision followup ends. This clinical feature poses unique challenges for standard clinical followup techniques.

In comparing NAER and published cases studies, we attribute differences in ethnicity and percent resulting from circumcision to the scope of the NAER dataset, which, like the VMMC program itself, covers only sub-Saharan Africa. A smaller proportion of keloid cases from NAER (1/11) than cases from literature review (9/29) were in clients with known personal or familial keloid history. History screening in the VMMC program may have caused this difference by preventing additional cases in predisposed clients in the NAER dataset, but is also clearly unable to eliminate keloids entirely. Clinical features were broadly similar across data sources. The disuse of radiotherapy, compression and silicone gel or sheet occlusion in the NAER cases presumably reflects the limited availability noted above.

Our analysis had several limitations. While reported keloids were extraordinarily rare, the true ascertainment of NAE reporting is not known, particularly for such a late-developing complication. In fact, the predominance of some countries in NAERS cases may reflect differences in diagnosis and/or in reporting, rather than risk. Some variables of interest, such as suture type, were not captured. These can be sought during the investigation process for future NAER keloid reports. Others, such as clinical setting, were captured, but showed no obvious patterns and had no denominators. NAER data (and the VMMC program) is limited to sub-Saharan Africa.

Additionally, though steroid injections were frequently reported, type, strength, dosing frequency, total doses, and injection technique were largely missing. Some pertinent negative “findings” may in fact reflect limited ascertainment due to the time lapses. Follow-up was also too brief to reach conclusions about clinical outcomes.

Finally, statistical results must be interpreted cautiously. Low event numbers with high participant numbers can lend strong statistical significance to differences small enough to be unstable. While we believe a true difference in keloid risk between age groups is likely, conclusive proof would require more complete ascertainment than NAER can provide.

Despite these limitations, the NAER dataset is unique in its size, its prospective nature, and its resulting fitness for statistical testing, for a complication on which literature has previously been dominated by case studies and series.

## Conclusion

Keloids were an extraordinarily rare complication of circumcision, and were more common after circumcisions performed in young adolescents. No keloids have been reported after PEPFAR largely discontinued circumcising clients less than 15 years old in 2020. Silicone gels or sheeting are a common management recommendation that could be added to current VMMC approaches with minimal risk. It is beneficial for keloid management to continue to include referral to an experienced physician and a conservative early approach. Personal and family keloid history screening to exclude those screening positive from VMMC remains important but cannot eliminate keloids; ensuring proper surgical technique (avoiding rough tissue handling and excessive wound tension) is already a best practice and may also decrease risk. Future NAE investigations can collect data on additional variables that may help refine keloid prevention and management.

## Supplementary Information

Supplementary Material 1.

## Data Availability

All relevant data for interpretation and replication is shared in the manuscript text and tables.

## Abbreviations

NAE: Notifiable adverse event
NAER: Notifiable adverse event reporting
PEPFAR: U.S. President’s Emergency Plan for AIDS Relief
VMMC: Voluntary medical male circumcision

## Footnotes

1 In addition to “freehand” surgical techniques, VMMC can also be conducted using WHO-prequalified surgical assist devices. One such device, the ShangRing, has a mechanism of action which protects against glans and shaft injuries. PEPFAR policy therefore permits circumcision of boys aged 13 years and older when using this device. In practice, its use has remained rare.

2 This date restriction was necessary because before 2015, NAE reporting was not mandatory and VMMC age data had substantial missingness. Additionally, 2023 was the year the last keloid NAE was reported; using it as a cutoff also allows sufficient time for keloids resulting from 2023 procedures to have developed.
