# Supplementary material for: Keloids as a rare complication of voluntary medical male circumcision: findings from PEPFAR’s notifiable adverse events reporting system
Source: BMC Urol. 2026 Jan 16;26:42. doi: 10.1186/s12894-025-01949-7 (PMC12892524; doi:10.1186/s12894-025-01949-7)
Supplement: Supplementary file 1 — Supplementary Material 1. [file 12894_2025_1949_MOESM1_ESM.docx]

**Supplementary online appendix: complete search strategies**

*Keloid epidemiology:*

| **Database** | **Strategy** | **Run Date** | **Records** |
| --- | --- | --- | --- |
| **Medline**  **(OVID)**  **1946-** | exp Cicatrix/ OR (keloid* OR (hypertroph* ADJ3 scar*)).ti,ab,kf.  AND  Circumcision, Male/ OR (circumci* AND (male* OR m?n OR boy*)).ti,ab,kf. | 2/21/2024 | 110  -  duplicates  = 109  unique items |
| **Global Health**  **(OVID)**  **1910-** | (keloid* OR (hypertroph* ADJ3 scar*)).ti,ab,hw.  AND  Circumcision, Male/ OR (circumci* AND (male* OR m?n OR boy*)).ti,ab,hw. |  | 6  -  duplicates  = 4  unique items |
| **CINAHL**  **(Ebsco)** | TI,AB,SU((MH "Cicatrix+") OR keloid* OR (hypertroph* N3 scar*))  AND  TI,AB,SU((MH "Circumcision") OR (circumci* AND (male* OR m?n OR boy*))) |  | 5  -  duplicates  = 1  unique items |
| **Scopus** | TITLE-ABS-KEY ( keloid* OR ( hypertroph* W/3 scar* ) ) AND TITLE-ABS-KEY ( circumci* AND ( male* OR m?n OR boy* ) ) |  | 48  -  duplicates  = 22  unique items |
| **Africa-Wide Information**  **(Ebsco)** | TI,AB(keloid* OR (hypertroph* N3 scar*))  AND  TI,AB(circumci* AND (male* OR m?n OR boy*)) |  | 2  -  duplicates  = 0  unique items |
| **African Index Medicus (AIM)**  **1975-** | TI,AB,SU((keloid* OR (hypertroph* AND scar*)))  AND  TI,AB,SU((circumci* AND (male* OR m?n OR boy*))) |  | 2  -  duplicates  = 1  unique items |

Notes: Duplicates were identified using the Endnote automated "find duplicates" function with preference set to match on title, author and year, and removed from your Endnote library. There will likely be additional duplicates found that Endnote was unable to detect.

*Keloid treatment:*

| **Database** | **Strategy** | **Run Date** | **Records** |
| --- | --- | --- | --- |
| **Medline**  **(OVID)**  **1946-** | exp Cicatrix/ OR (keloid* OR (hypertroph* ADJ3 scar*)).ti,ab,kf.  AND  Penis/ OR (penis* OR penile*).ti,ab,kf.  AND  (manage* OR treat* OR recommend* OR guideline* OR approach* OR prevent* OR outcome*).ti,ab,kf.  limit to yr="2014 -Current" | 02/21/2024 | 50 |
| **Global Health**  **(OVID)**  **1910-** | (keloid* OR (hypertroph* ADJ3 scar*)).ti,ab,hw.  AND  Penis/ OR (penis* OR penile*).ti,ab,hw.  AND  (manage* OR treat* OR recommend* OR guideline* OR approach* OR prevent* OR outcome*).ti,ab,hw.  limit to yr="2014 -Current" |  | 2  -  duplicates  = 0  unique items |
| **CINAHL**  **(Ebsco)** | TI,AB,SU((MH "Cicatrix+") OR keloid* OR (hypertroph* N3 scar*))  AND  TI,AB,SU((MH "Penis") OR penis* OR penile*)  AND  TI,AB,SU(manage* OR treat* OR recommend* OR guideline* OR approach* OR prevent* OR outcome*)  Publication Date: 2014-2024 |  | 5  -  duplicates  = 1  unique items |
| **Scopus** | TITLE-ABS-KEY ( keloid* OR ( hypertroph* W/3 scar* ) ) AND TITLE-ABS-KEY ( penis* OR penile* ) AND TITLE-ABS-KEY ( manage* OR treat* OR recommend* OR guideline* OR approach* OR prevent* OR outcome* ) AND PUBYEAR > 2013 AND PUBYEAR < 2025 |  | 34  -  duplicates  = 19  unique items |
| **Africa-Wide Information**  **(Ebsco)** | TI,AB(keloid* OR (hypertroph* N3 scar*))  AND  TI,AB(penis* OR penile*)  AND  TI,AB(manage* OR treat* OR recommend* OR guideline* OR approach* OR prevent* OR outcome*) |  | 1  -  duplicates  = 0  unique items |
| **WHO Global Index Medicus**  **1975-** | TI,AB,SU(keloid* OR (hypertroph* AND scar*))  AND  TI,AB,SU(penis* OR penile*)  AND  TI,AB,SU(manage* OR treat* OR recommend* OR guideline* OR approach* OR prevent* OR outcome*)  Date: 2014 - 2024 |  | 1  -  duplicates  = 0  unique items |

Notes: Duplicates were identified using the Endnote automated "find duplicates" function with preference set to match on title, author and year, and removed from your Endnote library. There will likely be additional duplicates found that Endnote was unable to detect.
